# How populist-aligned views affect receipt of non-COVID-19-related public health interventions: a systematic review of quantitative studies

**DOI:** 10.1186/s12889-025-23265-3

**Published:** 2025-06-04

**Authors:** Kaitlin Conway-Moore, Jack M. Birch, Alison R. McKinlay, Fiona Graham, Emily Oliver, Clare Bambra, Michael P. Kelly, Chris Bonell

**Affiliations:** 1 NIHR Policy Research Unit Behavioural and Social Sciences, Department of Public Health, Environments and Society, London School of Hygiene and Tropical Medicine, London, WC1H 9SH UK; 2 NIHR Policy Research Unit Behavioural and Social Sciences, Population Health Sciences Institute, Faculty of Medical Sciences, Newcastle University, Newcastle Upon Tyne, UK; 3 Department of Clinical, Educational and Health Psychology, NIHR Policy Research Unit Behavioural and Social Sciences, Centre for Behaviour Change, University College London, London, UK; 4 Department of Public Health and Primary Care, Cambridge Public Health, University of Cambridge, Cambridge, UK

**Keywords:** Populism, Populist Attitudes, Public Health Interventions, Systematic Review, Quantitative Evidence

## Abstract

**Background:**

Globally, there is increasing evidence of resistance to government-led public health interventions in areas such as vaccination, climate change mitigation, sexual and reproductive healthcare, and the implementation of non-pharmaceutical infection control measures. One potential explanation for this could be the documented global rise in populist attitudes, characterised by distrust of scientific, government and other perceived ‘elites.’ While the effect of such attitudes on engagement with COVID-19-related interventions has been extensively considered and researched, their association with the receipt of other public health interventions is currently underexplored.

**Methods:**

To understand how populist-aligned views might influence the receipt of public health interventions addressing areas other than COVID-19, we systematically reviewed quantitative research published across thirteen bibliographic databases and relevant websites between 2008 and 2024. All studies were set in member countries of the Organisation for Economic Co-operation and Development (OECD).

**Results:**

Across 30 included studies, the vast majority of which were cross-sectional, we found evidence that populist-aligned attitudes have a negative impact on the receipt of public health interventions including vaccinations, sexual and reproductive health care and preventive health care. We also found preliminary evidence of the negative role of populist-aligned attitudes on the receipt of disease screening related to HIV/AIDS and adherence to non-pharmaceutical interventions during times of public health emergency, such as the 2009 H1N1 pandemic.

**Conclusions:**

Although providing limited evidence of causality, the findings from this review suggest the need for future policy in many OECD countries to focus on trust-building between the public and political, scientific, and medical establishments. They also indicate the need for mitigation strategies to overcome the potentially negative impact of populist-style hostility towards out-groups on attitudes related to pressing public health issues such as abortion and family planning, for example by drawing on empathy-centred approaches.

**Systematic review registration:**

PROSPERO registration number CRD42024513124.

**Supplementary Information:**

The online version contains supplementary material available at https://doi.org/10.1186/s12889-025-23265-3.

## Background

In recent years, there has been increasing backlash against public health interventions in countries around the world. To date, such backlash has been largely targeted towards vaccination campaigns, efforts to mitigate the health effects of climate change, access to sexual and reproductive healthcare, and the implementation of non-pharmaceutical infection control measures, the latter of which was especially seen during the COVID-19 pandemic [1–5]. Some of the strongest evidence related to the potential impact of this kind of backlash came in 2019 when the World Health Organization (WHO) declared vaccine hesitancy among the top ten threats to health globally [6]. Further evidence has been published since, and vaccine hesitancy has also been linked to a major measles outbreak in the United States in 2025, a disease which was considered eliminated in the country as of 2000 [7].

One potential driver for increased resistance to government-led public health interventions could be the rise in populist politics and movements that has taken place in many countries over the last several years [1]. Populism as a political movement draws on and fuels popular dissatisfaction with and/or alienation from mainstream government, and is generally constructed in terms of ‘the people’ standing in opposition to an ‘elite’ seen as depriving the people of their sovereignty and freedom, and/or pushing for unwelcome social change which favour elites or, in right-wing populism, minorities and ‘social pluralism’ [8–11]. As a result, populist movements are often linked to opposition towards government interventions and regulation, a rolling-back of diversity, equity and inclusion (DEI) initiatives, and a push for unrestricted majoritarian sovereignty [12].

While who is considered to be part of ‘the people’ and who constitute ‘the elite’ within populist movements can vary across time and the political spectrum, what matters most is the clear distinction of ‘us’ versus ‘them.’ In left-wing populist movements, this distinction is largely binary, with those economically in the middle and bottom of society viewed as pushing against those at the top. In right-wing populist movements, the distinction is more often triadic, with ‘the people’ seen in opposition to one or more ‘elite’ groups (i.e., government, businesses, scientists and the media, among others), as well as various ‘other’ or ‘out groups,’ (i.e., women, migrants, and racial, ethnic, sexual, gender, linguistic or religious minorities) [13, 14].

Several recent global processes and events are seen to have fuelled populist movements in countries around the world, including the rise of neoliberal governance strategies, globalisation, anxiety over immigration, war and climate change, and the COVID-19 pandemic [15]. As such, populism has been described as a’thin’ ideology, focused on mobilising political support to achieve the various social, political and economic goals of politicians who are often themselves members of the elite, rather than a ‘thick’ ideology with a clear and consistent set of beliefs or aims [15].

Although much of the discourse on populist politics and movements in recent years has focused on their negative effects on unity and liberalism within society, understanding the role of populist attitudes in the acceptability and uptake of public health interventions can be seen as a critical component in ensuring the ongoing and future success of such interventions. While the effects of populist-aligned views on public health interventions addressing COVID-19 are widely appreciated, and indeed have been documented in evidence syntheses by the present study’s authors, such views might also affect other areas of public health engagement, a relationship that is not well understood at present [16–18]. Nonetheless, an international evidence base on this topic is emerging and merits synthesis to inform how such interventions might be better designed or presented to reduce populist-informed opposition. Recognising that the roots and consequences of populism are very different in high-, middle- and low-income countries, [11] and that cultural, structural and political systems are a key context for delivering and engaging with public health interventions that can improve population health, we focus here solely on high-income countries defined as those belonging to the Organisation for Economic Co-operation and Development (OECD).

To this end, in this paper, we report on a synthesis of quantitative evidence to address the following research question: what is the association between populist-aligned views and attitudes towards and/or engagement with public health interventions addressing health topics other than COVID-19 among people living in OECD countries? This synthesis was conducted as part of a larger systematic review which brought together both qualitative and quantitative evidence on the ways populist-type views are linked to attitudes towards and engagement with public health interventions mainly related to COVID-19. While the overall aim of this work is to draw on a wide body of international evidence to inform public health planning in England, this synthesis and its overall implications will focus on the role of populist attitudes in the receipt of public health interventions on an international scale.

## Methods

Our systematic review was conducted to align with the Preferred Reporting Items for Systematic Reviews and Meta-Analyses (PRISMA) reporting guidelines [19] (Appendix 1). The review protocol was prospectively registered with PROSPERO (registration number CRD42024513124) and published in 2024 [20].

### Inclusion and exclusion criteria

This paper reports on evidence from quantitative studies on public health interventions other than those deployed in response to the COVID-19 pandemic. All these studies were included in a wider systematic review on the ways in which populist-aligned attitudes are associated or linked with the receipt of public health interventions, which included both qualitative and quantitative evidence. As such, we report here inclusion criteria for the overall review as well as specifically for the evidence synthesised in this paper. Given the focus on populist-aligned attitudes as our exposure measure of interest, we limited our review to literature published since 2008, the year of the global financial crisis, which has been seen as a key driver of the global rise in populist attitudes, and as such the year from which most of the contemporary literature on populism begins [21].

Studies also needed to focus on at least one existing (i.e., non-hypothetical) public health intervention, including but not limited to: vaccination; disease screening; non-pharmaceutical infection control; sexual/reproductive health care; increased access to health care; climate change mitigation; road safety; anti-pollution measures; water fluoridation; gun control; mental health care; promotion of healthy diet and exercise; and interventions related to gambling, tobacco, alcohol or drug use.

Further, studies needed to include an exposure measure(s) which aligned with the attitudes commonly associated with populism in the research literature on this topic, even if not explicitly utilised by the study authors as a measure of populist attitudes. This decision was made based on the idea that populism is a highly contested socio-political construct which might be used to imply a critical perspective on those holding such views [11]. As such, requiring included studies to explicitly use the term’populist’ or ‘populism’ might have biased our results. We therefore included studies where there was evidence of participants holding views that aligned with populism in the sense of being hostile towards (including lacking trust in) at least one of the following: 1) elites (e.g., government, business, medical and other health professionals, mainstream media, science, and the wealthy); 2) out-groups (e.g., women, migrants, minoritised ethnic/racial/religious groups or gender/sexual minorities); 3) checks on popular sovereignty (e.g., legal rights, personal freedoms, and other government-imposed regulations); or 4) social change (including moves towards greater social pluralism for example via promotion of DEI, state intervention, or market regulation).

Finally, included studies needed to report how populist-aligned attitudes were associated with the receipt of public health interventions (i.e., attitudes towards, adherence to and/or uptake of such interventions).

### Search strategy and study selection

Searches for eligible studies were executed in thirteen bibliographic databases, all relevant to medical, psychological, economic and social scientific research. These included: CINAHL; Dissertation Abstracts; Econlit; EMBASE; Global Health; Global Index Medicus; International Bibliography of the Social Sciences; Ovid MEDLINE; PsycINFO; Scopus; Social Policy and Practice; Sociological Research Online; and Web of Science (including Science Citation Index Expanded, Social Sciences Citation Index, Arts & Humanities Citation Index, and Emerging Sources Citation Index). Our search was not limited by language or publication type. Given the nature of our research question, these searches mainly included the use of free-text terms rather than controlled vocabularies such as medical subject headings (MeSH).

Three concepts were taken from our inclusion criteria to develop a search string that was deployed in each of the above databases: populist attitude AND public health intervention AND intervention receipt. For each concept, relevant free-text and, where applicable, controlled-vocabulary terms were linked by ‘OR.’ Final search terms were reviewed by a librarian at the London School of Hygiene & Tropical Medicine prior to search execution. Appendix 2 contains the search strategy used in each of our included databases.

In addition to database searches, searches were run on the following websites, based on their relevance to our topic of interest: Centres for Disease Control and Prevention; Community Research and Development Information Service; Drug and Alcohol Findings Effectiveness Bank; European Centre for Disease Prevention and Control; Google; Google Scholar; Intergovernmental Panel on Climate Change; International Planned Parenthood Federation; Marie Stopes International; The Campbell Library; Open Library; United Nations Environment Programme; and the World Health Organization.

Following title and abstract as well as full-text screening, reference lists of all studies included in the wider systematic review were hand-searched for additional studies that met our inclusion criteria. Lastly, we contacted subject experts for studies meeting our inclusion criteria that may not have already been found.

All results obtained from our searches were downloaded into EPPI-Reviewer 6, at which point duplicates were removed [22]. Pilot screening of titles and abstracts began among pairs of reviewers (comprising KCM together with either FG or CBo) who screened successive batches of 50 records and then met to discuss any disagreements, calling on a third reviewer where necessary. Once a batch-level agreement of 90% was achieved via pilot screening, remaining titles and abstracts were divided amongst the group and screened for potential inclusion by one reviewer (KCM, FG or CBo). The process for screening full texts aimed to use a similar approach. However, as a 90% batch-level agreement was never achieved in the pilot screening of full texts, all full texts were screened by pairs of reviewers (comprising KCM together with either AMK or CBo), with regular meetings to discuss disagreements and consultation with a third reviewer where necessary.

### Data extraction and quality assessment

Details of all included studies were extracted by one reviewer (KCM) using Microsoft Excel, which were then cross-checked by a second reviewer (JB) [23]. Given the large volume of included studies, including a significant number of cross-sectional studies conducted on the topic of COVID-19, the decision was made to deviate from the study protocol by conducting a basic mapping of all included studies (i.e., describing basic study details, methods, sample and outcome measures) and then only synthesising the findings from: 1) all qualitative studies; 2) all longitudinal, quantitative studies (most of which focused on COVID-19); and 3) all quantitative studies on topics other than COVID-19, the latter of which are reported in this paper. This decision enabled us to focus our synthesis on studies providing the strongest qualitative insights into people’s lived experiences, quantitative evidence of how holding populist-aligned views might affect the receipt of public health interventions addressing COVID-19, and quantitative evidence describing how populist-type views are associated with engagement with public health interventions addressing health topics other than COVID-19. We included both cross-sectional and longitudinal evidence in the last of these syntheses given the emerging nature of the evidence base and the value of cross-sectional evidence in describing patterns of engagement.

Data extraction from the studies included in this evidence synthesis was carried out by one reviewer (KCM) using Microsoft Word, with cross-checking by a second reviewer (JB) [24]. This extraction followed the review protocol by reporting: basic study details (i.e., first author, publication date, study location, duration and timing of outcome measurements (if applicable)); study methods (i.e., design, sampling and sample size, participant characteristics, control for confounding, analytical approach and association between populist-aligned attitudes and public health intervention measured); and results (i.e., metric of association, estimate of association and *p*-value/confidence interval).

The Cambridge Quality Checklists were used to assess the quality of studies, with the three checklists considering correlates, risk factors and causal risk factors included within each study, respectively [25]. Specifically, the correlate checklist included questions related to sampling, sample size, response rate, measure of correlate and measure of outcome (for a total correlate score out of 5); the risk factor checklist assessed the use of cross-sectional, retrospective or prospective data (for a total risk factor score out of 3); and the causal risk factor checklist assessed variation in the risk factor, risk factor balancing and analysis of change (for a total causal risk factor score of 7) [25]. Quality assessment was carried out independently by two reviewers (KCM and JB) who met to compare their assessments, discuss any disagreements and reach an agreed score. The strength of the evidence presented in our synthesis for each outcome was assessed using the Grading of Recommendations, Assessment, Development, and Evaluations (GRADE) framework [26]. This takes into account both the study design and other factors, rating observational studies as relatively weak, downgrading evidence where there is risk of bias, inconsistency of evidence, lack of pertinence to population, imprecision and publication bias, but upgrading evidence where there are large effects, dose–response gradients or where any residual confounding would likely add to associations. Initial GRADE assessments were made by one reviewer (KCM), with cross-checking for accuracy by a second reviewer (JB).

### Data analysis and synthesis

Data from the results sections of each study were extracted into a Microsoft Word table by one reviewer (KCM), with cross-checking for accuracy by a second reviewer (JB) [24]. Results were then synthesised narratively and grouped according to the health topic of interest, such as vaccination, sexual and reproductive health care, preventive health care, disease screening and non-pharmaceutical infection control measures.

### Patient and public involvement

As a study funded by the National Institute for Health and Care Research (NIHR) Policy Research Unit Behavioural and Social Sciences, this review was supported by our designated patient and public involvement and engagement (PPIE) strategy group which has been regularly engaged during the study to comment on our methods and emerging findings. The overall goal of this collaboration is to improve the relevance of our work to a wide audience.

## Results

### Overview of included studies

**Fig. 1:**
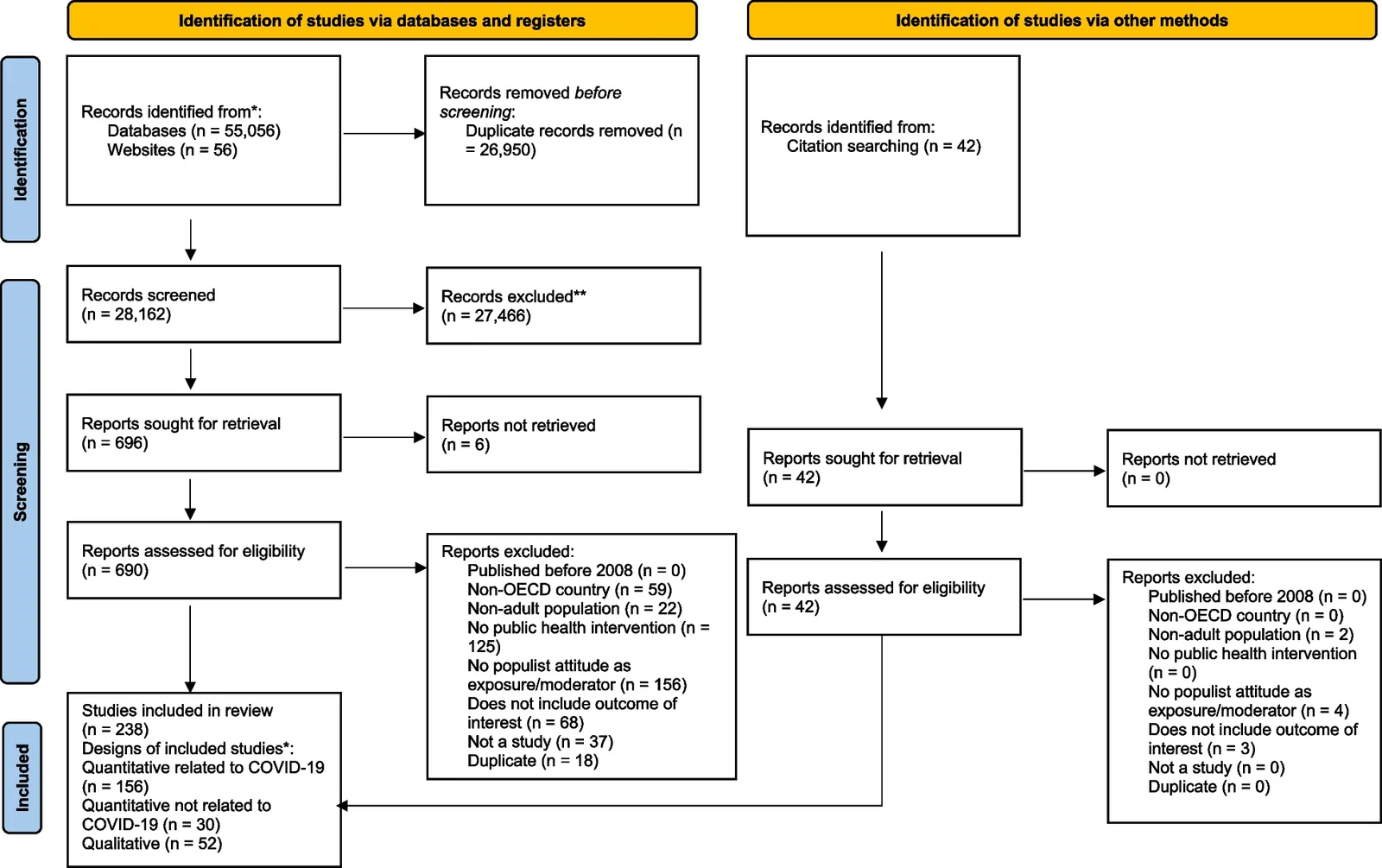
Preferred Reporting Items for Systematic Reviews and Meta-Analyses (PRISMA) flow diagram [27]. *The breakdown of included studies by study design totals to *n* = 246 rather than *n* = 238 as 8 studies included mixed methods

Figure 1 presents the results of our search strategy. Overall, we identified 55,056 references from database searches and 56 references from website searches, for a total of 55,112 references. After de-duplication, 28,162 unique references remained, which were further reduced to 690 after title and abstract screening. An additional six references were eligible for full-text screening but could not be retrieved. We identified 205 references for inclusion based on full-text screening, with an additional 33 references added based on hand-searching the reference lists of these included studies. Expert consultation identified no additional studies. This process resulted in a total of 238 references, of which 30 are included in the present review of non-COVID-19-focused quantitative studies.

### Study characteristics

All included studies were published between 2008 and 2023, with most published from 2018 onwards (Table 1).^1^ Study settings ranged geographically and included the United States (*n* = 17), Italy (*n* = 3), Poland (*n* = 3), Australia (*n* = 2), Japan (*n* = 2), Austria (*n* = 1), Germany (*n* = 1), Sweden (*n* = 1), Switzerland (*n* = 1) and Mexico (*n* = 1) (does not add to 30 since some studies international). Most studies (*n* = 28) utilised a cross-sectional study design, with two studies using a longitudinal design. Sampling methods included nationally representative sampling (*n* = 11), convenience sampling (*n* = 10), purposive sampling (*n* = 6), random sampling (*n* = 4) and time-location or venue-based sampling (*n* = 1 each). Sample sizes varied from 113 participants to as many 26,313, while also covering a range of participant characteristics such as parents (*n* = 6), HIV-positive adults (*n* = 1), male clients of female sex workers (*n* = 1), racial/ethnic minorities (*n* = 4), senior citizens (*n* = 1), and adult members of the general population of the study setting (*n* = 19).

**Table 1:** Included study characteristics and results (*n* = 30)

Study author (year)	Study country (city or region) and duration	Design, sampling method, sample size and participant characteristics	Adjustment	Analysis method and metric of association	Association measured	Estimate of association	*P* value/confidence interval for estimate of association
Aechtner and Farr (2022) [28]	Australia; 2018	Cross-sectional; Random sampling; 1,287 adult participants in the Australian Survey of Social Attitudes (AuSSA); 1,003 adult participants in the Wellcome Global Monitor (WGM)		Univariate linear regression, Beta coefficient	Association between confidence in Australia’s Federal Parliament and belief in childhood vaccines’ effectiveness in AuSSA dataset	0.161	95% CI: 0.001–0.002, *p* < 0.000
					Association between trust in scientists and belief in vaccine safety among WGM respondents	0.110	95% CI: 0.035–0.269, p ≤ 0.01
					Association between trust in scientists working for companies to be open and honest about who is paying for their work and belief in vaccine safety among WGM respondents	0.083	95% CI: 0.003–0.196, *p* = 0.043
Baumgaertner (2018) [29]	United States; January 2017	Cross-sectional; Nationally representative; 1,006 adult respondents from Survey Sampling International	Adjustment for age, race/ethnicity, education and income	Structural equation modelling; Beta coefficient	Direct/total association between trust in health care provider and attitudes towards pertussis, measles, and influenza vaccination	0.27	p ≤ 0.05
					Direct/total association between trust in government medical experts and attitudes towards pertussis, measles, and influenza vaccination	0.19	p ≤ 0.05
Bianco (2019) [30]	Italy (Catanzaro and Cosenza regions); April-June 2017	Cross-sectional; Random sample; 575 parents of children aged 1–5 years attending kindergarten in the geographic area of Catanzaro and Cosenza, southern Italy	Adjustment for gender, age, marital status, level of education, working activity, and nationality of the parent	Multivariable logistic regression; Adjusted odds ratio (AOR)	Belief that infant vaccinations are primarily a money-making operation for pharmaceutical companies on child vaccination hesitancy	Uncertain (as compared to disagree): 0.35	95% CI: 0.05–2.58, *p* = 0.306
						Agree (as compared to disagree): 2.66	95% CI: 0.59–11.95, *p* = 0.203
					Belief that infant vaccinations are primarily a money-making operation for pharmaceutical companies on child vaccination refusal	Uncertain (as compared to disagree):	N/A (removed from the model)
						Agree (as compared to disagree): 1.59	95% CI: 1.01–2.51, *p* = 0.045
					Parental trust in the paediatrician regarding received information about vaccines on child vaccination refusal	Yes (as compared to no): 0.56	95% CI: 0.32–0.96, *p* = 0.036
Börjesson (2014) [31]	Sweden; April-August 2010	Cross-sectional; Random sample; 1,587 Swedish adults	Adjustment for sex, age, working status, annual income, educational level, having children aged 0–6 in the household and belonging to a risk group	Hierarchical logistic regression; Odds ratio (OR)	Trust in authorities to handle the 2009 H1 N1 outbreak on vaccine uptake	1.71	95% CI: 1.30–2.25, *p* = 0.00
Cizmar (2023) [32]	United States; 2012, 2016 and 2020	Cross-sectional; Nationally representative; 11,424 US voters in 2012; 4,270 US voters in 2016; and 15,729 US voters in 2020, all based on data from the American National Election Studies (ANES)	Adjustment for gender, race/ethnicity, sexual orientation, marital status, perceptions of discrimination against women in the US, political affiliation, political ideology, religion, religiosity	Multinomial logistic regression; Coefficient estimates	Association between hostile sexism and abortion attitudes in 2012: Support if clear need (reference: Purely pro-choice)	0.34	SE: 0.36
					Association between hostile sexism and abortion attitudes in 2012: Support in the case of rape, incest, woman’s health (reference: Purely pro-choice)	1.07	SE: 0.31, *p* < 0.001
					Association between hostile sexism and abortion attitudes in 2012: Purely pro-life (reference: Purely pro-choice)	1.09	SE: 0.41, *p* < 0.01
					Association between hostile sexism and abortion attitudes in 2016: Support if clear need (reference: Purely pro-choice)	2.00	SE: 0.46, *p* < 0.001
					Association between hostile sexism and abortion attitudes in 2016: Support in the case of rape, incest, woman’s health (reference: Purely pro-choice)	2.77	SE: 0.41, *p* < 0.001
					Association between hostile sexism and abortion attitudes in 2016: Purely pro-life (reference: Purely pro-choice)	2.80	SE: 0.53, *p* < 0.001
					Association between hostile sexism and abortion attitudes in 2020: Support if clear need (reference: Purely pro-choice)	0.77	SE: 0.46, *p* < 0.10
					Association between hostile sexism and abortion attitudes in 2020: Support in the case of rape, incest, woman’s health (reference: Purely pro-choice)	1.15	SE: 0.40, *p* < 0.01
					Association between hostile sexism and abortion attitudes in 2020: Purely pro-life (reference: Purely pro-choice)	1.97	SE: 0.63, *p* < 0.01
					Association between hostile sexism and predicting pure pro-life vs pure pro-choice in 2012	0.80	SE: 0.43, *p* < 0.10
					Association between hostile sexism and predicting pure pro-life vs pure pro-choice in 2016	2.26	SE: 0.59, *p* < 0.001
					Association between hostile sexism and predicting pure pro-life vs pure pro-choice in 2020	1.94	SE: 0.73, *p* < 0.01
Clark (2008) [33]	United States (Houston); March-December 2005	Cross-sectional; Convenience sample; 113 HIV-positive adult patients (diagnosed in the last three years) in four public health facilities		Univariate statistical analysis; Mean/Standard Deviation (SD)	Mean HIV conspiracy belief score among those who have never received HAART	M: 16.2, SD: 7.1	*p* = 0.78
					Mean HIV conspiracy belief score among those who have received HAART	M: 16.7, SD: 6.6	
					Mean HIV conspiracy belief score among those not currently on HAART	M: 16.1, SD: 6.8	*p* = 0.71
					Mean HIV conspiracy belief score among those currently on HAART	M: 16.8, SD: 6.7	
					Mean HIV conspiracy belief score among those with < 100% adherence to HAART by self-report	M: 16.4, SD: 6.9	*p* = 0.78
					Mean HIV conspiracy belief score among those with 100% adherence to HAART by self-report	M: 15.9, SD: 7.0	
					Mean HIV conspiracy belief score among those with < 80% adherence to HAART by pharmacy refill data	M: 18.8, SD: 6.8	*p* = 0.18
					Mean HIV conspiracy belief score among those without < 80% adherence to HAART by pharmacy refill data	M: 15.4, SD: 7.2	
					Mean HIV conspiracy belief score among those with a gap in care > 120 days	M: 16.9, SD: 7.0	*p* = 0.91
					Mean HIV conspiracy belief score among those without a gap in care > 120 days	M: 17.1, SD: 6.8	
			Adjustment for race/ethnicity and time since HIV diagnosis	Multivariable regression analyses	Association between HIV conspiracy beliefs and: those who have never received HAART; those not currently on HAART; those with < 100% adherence to HAART by self-report; those with < 80% adherence to HAART by pharmacy refill data; and those with a gap in care > 120 days	Data not provided	All *p* = 0.13
Fleming (2017) [34]	United States and Mexico (San Diego and Tijuana); September 2010-October 2012	Cross-sectional; Time-location sample; 400 adult male clients of female sex workers (FSWs); participants were enrolled in a sexual risk reduction intervention known as Hombre Seguro (‘‘Safe Men’’), half from San Diego and half from Tijuana	Adjustment for age, education	Multiple logistic regression; Adjusted odds ratio (AOR)	Association between misogyny scale score and ever having tested for HIV	0.39	95% CI: 0.13–1.14, *p* = 0.09
			Adjustment for age	Backwards stepwise multiple logistic regression; Adjusted odds ratio (AOR)	Association between misogyny scale score and ever having tested for HIV	0.31	95% CI: 0.11–0.84, *p* = 0.02
Ford (2013) [35]	United States (Los Angeles); August 2006-May 2007	Cross-sectional; Random sample; 226 adult participants, aged 50 + and participating in the LA VOICES study, which includes socially vulnerable, racially/ethnically diverse men and women living in Los Angeles	Adjustment for sex, race/ethnicity, education, risk category, AIDS conspiracy belief and place of usual care	Backwards stepwise multiple logistic regression; Adjusted odds ratio (AOR)	Association between mistrust in government and HIV testing in the last 12 months	0.43	95% CI: 0.26–0.73
Frew (2012) [36]	United States (Atlanta); September-December 2009	Cross-sectional; Venue-based random sample; 503 US adults from predominantly racial/ethnic minority backgrounds; recruited from churches, bookstores, educational forums, community meetings, and special events such as family health fairs	Adjustment for education and income	Multivariate logistic regression; Odds ratio (OR)	Association between a scale measuring conspiracy beliefs about H1 N1/mistrust of H1 N1 information coming from the government and intention to receive an H1 N1 vaccine	1.63	1.13, 2.35, *p* < 0.05
					Association between a scale measuring conspiracy beliefs about H1 N1/mistrust of H1 N1 information coming from the government and intention to receive seasonal flu vaccine	1.64	95% CI: 1.23–2.19, *p* < 0.05
Frietze (2023) [37]	United States (El Paso, Texas); June–August 2020	Cross-sectional; Purposive sample; 602 predominantly Hispanic adults living in the US-Mexico border town of El Paso, Texas		Logistic regression; Odds ratio (OR)	Trust in government related to vaccines and HPV vaccine uptake	1.06	95% CI: 0.88–1.28
				Linear regression; Beta coefficient	Trust in government related to vaccines and HPV vaccine intention	0.31	95% CI: 0.22–0.43, *p* < 0.001
Gilles (2011) [38]	Switzerland; March 2009-December 2009 (follow-up 6 months after baseline)	Longitudinal; Convenience sample; 601 French-speaking Swiss adults	Adjustment for age, gender, residential area, education, income, and number of children	Logistical hierarchical regression; Beta coefficient	Trust in medical organisations on vaccine uptake	0.76	SE: 0.21; *p* < 0.001
					Trust in medical organisations on vaccine uptake	0.30	SE: 0.08; *p* < 0.001
				Hierarchical linear regression; Beta coefficient	Trust in medical organisations on perceived efficacy of washing hands	0.17	SE: 0.06; *p* < 0.01
					Trust in medical organisations on perceived efficacy of wearing a mask	0.22	SE: 0.08; *p* < 0.01
Gothreau (2022) [39]	United States: Winter 2018	Cross-sectional; Study 1: Nationally representative sample; 1,400 US adults	Adjustment for age, gender, income, education, religiosity, race and ideology	Ordinary Least Squares (OLS) regression; Regression coefficient	Association between benevolent sexism and attitudes towards abortion	− 0.068	SE: 0.026, *p* < 0.05
					Association between hostile sexism and attitudes towards abortion	− 0.080	SE: 0.024, *p* < 0.01
					Association between benevolent sexism and attitudes towards women’s access to birth control	− 0.017	SE: 0.021
					Association between hostile sexism and attitudes towards women’s access to birth control	− 0.205	SE: 0.020, *p* < 0.01
					Association between benevolent sexism and attitudes towards federal funding for Planned Parenthood	− 0.069	SE: 0.035, *p* < 0.10
					Association between hostile sexism and attitudes towards federal funding for Planned Parenthood	− 0.325	SE: 0.033, *p* < 0.01
		Cross-sectional; Study 2: Nationally representative; 4,270 adult respondents from the 2016 American National Election Study	Adjustment for age, gender, income, education, religiosity, race and ideology	Ordinary Least Squares (OLS) regression; Regression coefficient	Association between hostile sexism and attitudes towards abortion	− 0.129	SE: 0.030, *p* < 0.01
Hamada (2015) [40]	Japan (Fukuoka prefecture); November 2012-April 2013	Cross-sectional; Convenience sample; 1,407 mothers of daughters aged 13–16 years in two middle schools and ten high schools in Fukuoka prefecture		Bivariate logistic regression; Odds ratio (OR)	Association between mother’s trust in the government’s handling of vaccinations and daughter’s HPV vaccination status (reference: No trust in government)	4.49	95% CI: 3.17–6.37, *p* < 0.001
			Adjustment for educational background, annual household income, marital status and employment status	Multivariate logistic regression; Odds ratio (OR)	Association between mother’s trust in the government’s handling of vaccinations and daughter’s HPV vaccination status (reference: No trust in government)	2.40	95% CI: 1.44–3.86, *p* < 0.001
Hori (2023) [41]	Japan; September–October 2022	Cross-sectional; Purposive sample; 26,313 Japanese adults from the Japan COVID-19 and Society Internet Survey	Adjustment for age group, educational background, sexual orientation, working status, household income, COVID-19 vaccination status, and frequency of going to a brothel	Modified Poisson regression analysis; Prevalence ratios	Association between assigned men’s trust in government and mpox vaccine intention (as compared to no trust in government)	1.37	95% CI: 1.29–1.45
					Association between assigned women’s trust in government and mpox vaccine intention (as compared to no trust in government)	1.35	95% CI: 1.23–1.47
Kohler (2023) [42]	Germany and Austria; May–June 2021	Cross-sectional; Convenience sample; 870 German and Austrian adults	Adjustment for age, gender, education, health information seeking and health consciousness	Binomial logistic regression; Odds ratio (OR)	Association between science-related populism and measles, mumps, rubella (MMR) vaccination uptake	0.602	95% CI:0.49–0.72, *p* < 0.001
					Association between science-related populism and tick-borne encephalitis (TBE) vaccination uptake	0.884	95% CI: 0.76–1.02
					Association between science-related populism and human papillomavirus (HPV) vaccination uptake	0.988	95%CI: 0.81–1.20
					Association between science-related populism and seasonal influenza vaccination uptake	0.907	95% CI: 0.78–1.05
					Association between science-related populism and meningococcal disease (MD) vaccination uptake	0.833	95% CI: 0.71–0.97
Kossowska (2021) [43]	Poland; March 2020 and June 2020	Cross-sectional; Purposive sampling; Study 2: 391 Polish adults	Adjustment for age, gender, education, political knowledge and being afraid of COVID-19	Mediation analysis; Beta coefficient	Association between right-wing political ideology and trust in scientists	−0.14	95% CI: −0.22 – −0.06, *p* < 0.01
					Association between trust in scientists and positive attitudes towards vaccines	0.24	95% CI: 0.14–0.34, *p* < 0.001
					Indirect association between right-wing political ideology and positive attitudes towards vaccines via trust in scientists	−0.04	95% CI: −0.08 – −0.01
		Cross-sectional; Purposive sampling; Study 3: 376 Polish adults	Adjustment for age, gender, education, political knowledge and being afraid of COVID-19	Mediation analysis; Beta coefficient	Association between right-wing political ideology and perception of scientists as members of the country’s elite	0.27	95% CI: 0.20, 0.35, *p* < 0.01
					Association between perception of scientists as members of the country’s elite and positive attitudes towards vaccines	−0.21	95% CI: −0.33 – −0.10, *p* < 0.01
					Indirect association between right-wing political ideology on positive attitudes towards vaccines via perception of scientists as members of the country’s elite	−0.07	95% CI: −0.13 – −0.02
Krupenkin (2021) [44]	United States; October 2009 and April 2015	Cross-sectional; Nationally representative; H1 N1 data: 1,004 US adults; Measles data: 4,570 US adults	Adjustment for age, gender, race and education	Regression analysis; Regression coefficient	Association between feeling somewhat/very confident in the US government and attitudes towards the safety of the H1 N1 vaccine	1.359	SE: 0.852
					Association between feeling somewhat/not so confident in the US government and attitudes towards the safety of the H1 N1 vaccine	−0.763	SE: 0.852
					Association between feeling not so/not at all confident in the US government and attitudes towards the safety of the H1 N1 vaccine	−2.226	SE: 0.855, *p* < 0.01
					Association between feeling very/somewhat confident in the US government and attitudes towards the safety of the MMR vaccine	0.655	SE: 0.413
					Association between feeling somewhat/not very confident in the US government and attitudes towards the safety of the MMR vaccine	−1.412	SE: 0.413, *p* < 0.01
					Association between feeling not very/not at all confident in the US government and attitudes towards the safety of the MMR vaccine	−2.795	SE: 0.419, *p* < 0.01
Lee (2016) [45]	United States (Colorado, Massachusetts, Missouri and Washington); 2002–2003	Cross-sectional; Purposive sample; 1,253 parents of school children in four US states, who both had and didn't have a non-medical exemption for vaccinating their children	Adjustment for income, education, race, religiousness, and age	Bivariate logistic regression; Odds ratio (OR)	Association between parent not fully vaccinating their child and distrust in government (as compared to trust)	1.97	95% CI: 1.45–2.67, *p* < 0.01
					Association between parent not fully vaccinating their child and distrust in healthcare provider (as compared to trust)	2.18	95% CI: 1.63–2.92, *p* < 0.01
					Belief that immunizations do more harm than good and distrust in government (as compared to trust)	1.95	95% CI: 1.38–2.74, *p* < 0.01
					Belief that immunizations do more harm than good and distrust in healthcare provider (as compared to trust)	2.03	95% CI: 1.45–2.81, *p* < 0.01
Leonard (2015) [46]	United States; 2010	Cross-sectional; Purposive sampling; 243 parents with at least one 6-year-old child		Linear regression; R^2^	Association between general trust in medical authorities (i.e., doctors and the government) and attitudes towards MMR vaccine	R^2^ = 0.10, F(1, 235) = 26.39	*p* < 0.001
			Adjustment for gender	Moderated Multiple regression; ∆R^2^	Association between general trust in medical authorities (i.e., doctors and the government) and attitudes towards MMR vaccine, moderated by gender	∆R^2^ < 0.001, ∆F(1, 233) = 0.10	∆*p* = 0.75
			Adjustment for age		Association between general trust in medical authorities (i.e., doctors and the government) and attitudes towards MMR vaccine, moderated by age	∆R^2^ < 0.01, ∆F(1, 233) = 0.51	∆*p* = 0.29
			Adjustment for education		Association between general trust in medical authorities (i.e., doctors and the government) and attitudes towards MMR vaccine, moderated by education	∆R^2^ < 0.01, ∆F(1, 233) = 0.39	∆*p* = 0.53
Mesch (2015) [47]	United States; October 2009	Cross-sectional; Nationally representative sample; 968 US adults	Adjustment for age, sex, ethnicity, presence of children in the home, level of education, and social attitudes (i.e., liberalism or conservatism)	Logistic regression; Odds ratio (OR)	Trust in the federal government to handle an outbreak of H1 N1 on H1 N1 vaccine uptake	1.58	95% CI: 1.10–2.26, *p* < 0.01
					Trust in the local healthcare system to handle an outbreak of H1 N1 on H1 N1 vaccine uptake	1.60	95% CI: 1.04–2.45, *p* < 0.05
Musa (2009) [48]	United States (Allegheny County, Pennsylvania); June 2001-May 2002	Cross-sectional; Convenience sample; 1,681 Black and White adults aged 65 + who were enrolled in the Medicare Enrollment File (MEF) for Allegheny County	Adjustment for gender, age, education, marital status), self-reported health status and number of health conditions	Logistics regression; Odds ratio (OR)	Trust in formal health information source on uptake of flu shot in the past year	1.16	95% CI: 0.72–1.85
					Trust in one’s own doctor on uptake of flu shot in the past year	1.22	95% CI: 0.55–2.72
					Trust in formal health information source on PSA test for men in the last year	0.80	95% CI: 0.43–1.51
					Trust in one’s own doctor on PSA test for men in the last year	8.59	95% CI: 2.66–27.68, p ≤ 0.001
					Trust in formal health information source on mammogram for women in the last two years	0.73	95% CI: 0.32–1.67
					Trust in one’s own doctor on mammogram for women in the last two years	3.97	95% CI: 1.17–13.55, p ≤ 0.05
					Trust in formal health information source on routine check-up in the last year	0.70	95% CI: 0.33–1.50
					Trust in one’s own doctor on routine check-up in the last year	3.04	95% CI: 1.02–9.05, p ≤ 0.05
Ojikutu (2018) [49]	United States; February-April 2016	Cross-sectional; Nationally representative of the black population; 855 Black adults 18–50 years who were HIV negative	Adjustment for gender, age, income, education, employment, marital status, mental health (depressive symptoms), access to health care (insurance status and last appointment with a health care provider), alcohol use, HIV risk-related his- tory (sexual behavior in the 3 months prior to the survey and drug use-powder or crack cocaine, heroin, or crystal methamphetamine) and history of HIV testing	Bivariate logistic regression; Odds ratio (OR)	Trust in medical doctor completely or mostly compared to a little, not at all) on willingness to use PrEP among all participants	0.9	95% CI: 0.6, 1.3, *p* = 0.5536
					Trust in medical doctor completely or mostly compared to a little, not at all) on willingness to use PrEP among high risk participants	0.8	95% CI: 0.5–1.5, *p* = 0.5558
					HIV conspiracy beliefs on willingness to use PrEP among all participants	1.3	95% CI: 1.1–1.5, *p* = 0.0016
					HIV conspiracy beliefs on willingness to use PrEP among high risk participants	1.2	95% CI: 1.0–1.5, *p* = 0.1371
Powell (2019) [50]	United States (Michigan, Georgia, California, and North Carolina); 2003–2009	Cross-sectional; Convenience sample; 610 Black adults aged 20 + attending barbershops and two academic institutions/events	Adjustment for age, recruitment site type, region, education, income, marital status, health insurance status, usual source of care, self-rated health status, chronic conditions (hypertension, coronary heart disease, and any heart disease) and and depressive symptoms	Multiple logistic regression; Unadjusted odds ratios and adjusted odds ratios	Medical mistrust on preventive health screening delay: Routine Check up	UOR: 2.76	95% CI: 1.70, 4.47, *p* < 0.0001
					Medical mistrust on preventive health screening delay: Blood Pressure Screening	UOR: 2.50	95% CI: 1.49–4.19, *p* < 0.0001
					Medical mistrust on preventive health screening delay: Cholesterol Screening	UOR: 1.45	95% CI: 0.81–2.60, *p* = 0.22
					Medical mistrust only on preventive health screening delay: Routine Check up	AOR: 2.87	95% CI: 1.45–5.71, *p* < 0.001
					Medical mistrust only on preventive health screening delay: Blood Pressure Screening	AOR: 2.82	95% CI: 1.31- 6.05, *p* < 0.01
					Medical mistrust only on preventive health screening delay: Cholesterol Screening	AOR: 2.12	95% CI: 0.87- 5.17, *p* = 0.10
Prati (2011) [51]	Italy; February 2010	Cross-sectional; Nationally representative; 1,010 Italian adults	Adjustment for sex, age, work status, economic hardship and parental status	Multivariate logistic regression; Adjusted odds ratio (AOR)	Trust in the institutional response to the outbreak and stated willingness to comply with recommended behaviours: To clean objects	1.0	95% CI: 0.9–1.1
					Trust in the institutional response to the outbreak and stated willingness to comply with recommended behaviours: To wash hands	1.0	95% CI: 0.9–1.1
					Trust in the institutional response to the outbreak and stated willingness to comply with recommended behaviours: To use tissues when sneezing	1.1	95% CI: 1.0–1.2; *p* < 0.05
					Trust in the institutional response to the outbreak and stated willingness to comply with recommended behaviours: Social distancing	1.0	95%CI: 0.9–1.1
					Trust in the institutional response to the outbreak and stated willingness to comply with recommended behaviours: Vaccine acceptance	1.4	95% CI: 1.1–1.8. p < 0.05
					Media trust and stated willingness to comply with recommended behaviours: To clean objects	1.2	95% CI: 1.1–1.3, *p* < 0.05
					Media trust and stated willingness to comply with recommended behaviours: To wash hands	1.2	95% CI: 1.1–1.2, *p* < 0.05
					Media trust and stated willingness to comply with recommended behaviours and stated willingness to comply with recommended behaviours: To use tissues when sneezing	1.1	95% CI: 1.1–1.2, *p* < 0.05
					Media trust and stated willingness to comply with recommended behaviours: Social distancing	1.1	95% CI: 1.1–1.2, *p* < 0.05
					Media trust and stated willingness to comply with recommended behaviours: Vaccine acceptance	1.3	95% CI: 1.1–1.5, *p* < 0.05
					Trust in the Ministry of Health and stated willingness to comply with recommended behaviours: To clean objects	1.1	95% CI: 1.1–1.2, *p* < 0.05
					Trust in the Ministry of Health and stated willingness to comply with recommended behaviours: To wash hands	1.1	95% CI: 1.1–1.2, *p* < 0.05
					Trust in the Ministry of Health and stated willingness to comply with recommended behaviours: To use tissues when sneezing	1.1	95% CI: 1.0–1.1, *p* < 0.05
					Trust in the Ministry of Health and stated willingness to comply with recommended behaviours: Social distancing	1.1	95% CI: 1.0–1.1, *p* < 0.05
					Trust in the Ministry of Health and stated willingness to comply with recommended behaviours: Vaccine acceptance	1.4	95% CI: 1.1–1.6, *p* < 0.05
					Trust in medical science and stated willingness to comply with recommended behaviours: To clean objects	1.1	95% CI: 1.0–1.2, *p* < 0.05
					Trust in medical science and stated willingness to comply with recommended behaviours: To wash hands	1.0	95% CI: 0.9–1.1
					Trust in medical science and stated willingness to comply with recommended behaviours: To use tissues when sneezing	1.0	95% CI: 0.9–1.1
					Trust in medical science and stated willingness to comply with recommended behaviours: Social distancing	1.0	95% CI: 0.9–1.1
					Trust in medical science and stated willingness to comply with recommended behaviours: Vaccine acceptance	1.3	95% CI: 1.0–1.6, *p* < 0.05
Prusaczyk (2019) [52]	United States; March 2017	Cross-sectional; Convenience sample; 296 US adults recruited via Amazon Mechanical Turk	Adjustment for age, education and religiosity	Mediation analyses; Beta coefficient	The association between hostile sexism and support for abortion	‒0.11	95% CI: −0.23–0.01, *p* = 0.08
Quinn (2013) [53]	United States; January 2010	Cross-sectional; Nationally representative; 2,042 US adults	Adjustment for race/ethnicity, gender, age, education, income, political party, and political ideology	Sequential multinomial logistic regression; Relative Risk (RR)	Trust in government actions during the H1 N1 pandemic on H1 N1 vaccine uptake/intentions: Perception that government is concerned: Δ R^2^ = 0.01	Yes vs. No: 1.42	*p* > 0.05
						Don’t know vs. No: 1.40	*p* < 0.01
Selleri and Carugati (2020) [54]	Italy (Northern region); October 2016—March 2017	Cross-sectional; Convenience sample; 972 mothers of pre-school children aged 0–6	Adjustment for children’s age and gender, parents’ age, profession, educational level, number of children, confidence in healthcare authorities, relations between vaccines nature, universalistic values and social media use	Multiple correspondence analysis; Correlation coefficient	Association between belief in conspiracy theories about pharmaceutical companies and belief that “Vaccinating children is a private choice of parents: healthcare authorities do not have to intervene”	0.399	*p* < 0.001
					Association between trust in science and attitudes towards childhood vaccination	0.213	*p* < 0.001
Stasiuk (2021) [55]	Poland; February 2018-December 2020 (follow-up 34 months from baseline)	Longitudinal; Convenience sample; *n* = 400 Polish adults	Adjustment for sex, age, place of residence	Multiple linear regression; Beta coefficient	Trust in physicians on attitudes towards vaccination (in general) in 2018	0.228	SE: 0.061; *p* < 0.001
					Trust in physicians on attitudes towards vaccination (in general) in 2020	0.367	SE: 0.054; *p* < 0.001
					Trust in science on attitudes towards vaccination (in general) in 2018	0.3	SE: 0.053; *p* < 0.001
					Trust in science on attitudes towards vaccination (in general) in 2020	0.362	SE: 0.051; *p* < 0.001
					Change in trust in physicians between 2018 and 2020 on attitudes towards vaccination (in general) in 2020	0.244	SE: 0.059; *p* < 0.001
					Change in trust in scientists between 2018 and 2020 on attitudes towards vaccination (in general) in 2020	0.288	SE: 0.058; *p* < 0.001
					Trust in physicians as a predictor of attitudes towards COVID-19 vaccination in 2020	0.23	SE: 0.042; *p* < 0.05
					Trust in scientists as a predictor of attitudes towards COVID-19 vaccination in 2020	0.095	SE:0.040
Szczepańska (2022) [56]	Poland; November 2020 and December 2020	Cross-sectional; Nationally representative sample; Study 1: 994 Polish adults	Adjustment for gender, highest level of education and settlement size	Mediation analysis; Beta coefficient	Direct association of hostile sexism on support for abortion ban	Unstandardised β: 0.40	SE: 0.05; 95% CI: 0.29–0.50; *p* < 0.001
		Cross-sectional; Nationally representative sample; Study 2: 432 Polish adults	Adjustment for gender, highest level of education and settlement size		Direct association between benevolent sexism and support for abortion ban	Unstandardised β: –0.01	SE: 0.06; 95% CI[–0.12–0.10; *p* = 0.864
				Mediation analysis; Beta coefficient	Direct association between hostile sexism and support for abortion ban	Unstandardised β: 0.41	SE: 0.12; 95% CI: 0.17–0.64; *p* < 0.001
					Direct association between benevolent sexism and support for abortion ban	Unstandardised β: 0.01	SE: 0.12; 95% CI: −0.23–0.25; *p* = 0.927
					Direct association between prejudice towards people with Down syndrome and support for abortion ban	Unstandardised β: 0.32	SE: 0.15; 95% CI: 0.02- 0.61; *p* < 0.05
Wright (2023) [57]	Australia (Sunshine Coast, Queensland); 2020	Cross-sectional; Convenience sample; 1,050 parents aged 20–74	Adjustment for age, sex and education level	Hierarchical multiple regression; Beta coefficient	Trust in government to represent citizens and anti-vaccination attitudes	Unstandardised β: −0.22	SE: 0.02, *p* < 0.001
						Standardised β: −0.25	95% CI: 0.26–0.17

By way of exposure measures, most studies focused on populist-aligned attitudes as they related to perceptions of, and trust in, ‘elite’ institutions such as government, scientists, medical professionals, pharmaceutical companies and the media (*n* = 25), while five studies included measures of populist-aligned attitudes related to hostility towards out-groups, including women and people with disabilities. In terms of outcomes, the studies focused on a number of different types of public health interventions, including vaccination (*n* = 21), sexual and reproductive health care (*n* = 5), preventive health care (*n* = 3), disease screening (*n* = 2), and non-pharmaceutical infection control measures (*n* = 2).

### Quality of included studies and certainty of evidence

Based on the Cambridge Quality Checklists, the quality of the included studies was moderate [25]. Out of a possible score of 15 points (combining a correlate score out of 5, a risk factor score out of 3, and a causal risk factor score out of 7), six studies scored in the low-quality range with 4 points (*n* = 3) and 5 points (*n* = 3), respectively. Twenty-two studies scored in the medium-quality range with 6 points (*n* = 5), 7 points (*n* = 2), 8 points (*n* = 2), 9 points (*n* = 5), and 10 points (*n* = 8), respectively. Finally, two studies scored in the high-quality range, with 11 points and 12 points, respectively. A full breakdown of the quality assessment for each study can be found in Appendix 4. Based on the GRADE framework for certainty of evidence, the confidence in the findings of this review can be considered variable based on the outcome of interest. Of our five outcomes reported below (i.e., attitudes towards/uptake of: 1) vaccination; 2) sexual and reproductive health care; 3) preventive health care; 4) disease screening; and 5) non-pharmaceutical infection control measures) certainty in the evidence was determined to be moderate, moderate, very low, very low and very low, respectively. A full breakdown of the certainty of evidence for each of our reported outcomes can be found in Appendix 5.

### Narrative synthesis findings

Evidence from the 30 included studies was too heterogenous in terms of populations and measures for meta-analysis and so was subject to narrative synthesis, which is reported below. These findings have been organised according to the health topic under investigation in each study. We do not report size of associations for null results but these are reported in Table 1.

### Vaccination

Twenty-one studies examined the association between populist-aligned views and attitudes towards and/or uptake of vaccination, generally finding that such views were associated with decreased engagement with vaccination. Among the types of vaccination examined within these studies include H1 N1 (*n* = 7), general childhood vaccines (*n* = 4), human papillomavirus (HPV) (*n* = 3), measles, mumps, and rubella (MMR) (*n* = 3), seasonal influenza (*n* = 3), miscellaneous vaccines (*n* = 3) and attitudes towards vaccines in general (*n* = 4).

#### H1 N1

Seven studies examined the association between populist-aligned views and attitudes towards/uptake of the H1 N1 vaccine. A medium-quality study published in 2011 by Prati et al. [51] drew on a nationally representative sample of 1,010 Italian adults from early 2010 to examine associations between trust in several ‘elite’ institutions and acceptance of the H1 N1 vaccine. It was found that trust in the institutional response to H1 N1 was significantly associated with increased odds of vaccine acceptance (Adjusted Odds Ratio (AOR): 1.4, 95% CI: 1.1–1.8. *p* < 0.05), as was trust in the media, the Ministry of Health and medical science (AOR: 1.3, 95% CI: 1.1–1.5, *p* < 0.05; AOR: 1.4, 95% CI: 1.1–1.6, *p* < 0.05; and AOR: 1.3, 95% CI: 1.0–1.6, *p* < 0.05; respectively).

A medium-quality 2012 study by Frew et al. [36] sampled 503 predominantly racial/ethnic minority US adults in autumn 2009 to understand associations between a scale measuring conspiracy beliefs about H1 N1/mistrust in H1 N1 information coming from the government and intention to receive an H1 N1 vaccine. Controlling for education and income, it was found that lower scores on the conspiracy belief/government mistrust scale (indicative of less conspiracy beliefs and greater trust) was significantly associated with a greater intention to receive the H1 N1 vaccine (Odds Ratio (OR): 1.63, 95% CI: 1.13–2.35, *p* < 0.05).

A medium-quality study published in 2013 by Quinn et al. [53] used a nationally representative sample of 2,042 US adults from early 2010 to gage the impact of trust in government actions during the H1 N1 pandemic on vaccination intentions. It was found that responding ‘No’ or ‘Don’t know’ related to a perception that the government was concerned about the pandemic significantly and positively predicted vaccination intentions (Relative risk (RR): 1.40, *p* < 0.01), but that overall there was a minimal increase in predicting vaccination intention contributed by trust in government actions (Δ R^2^ = 0.01) compared to other variables included in their model.

A medium-quality 2014 study of 1,587 Swedish adults by Börjesson et al. [31] examined the association between trust in the Swedish authorities to handle the 2009 H1 N1 pandemic and vaccine uptake. Controlling for sex, age, working status, annual income, educational level, having children aged 0–6 in the household and belonging to a risk group, the study found that those with higher levels of trust in authorities were more likely to be vaccinated (OR: 1.71, 95% CI: 1.30–2.25, *p* = 0.00).

A medium-quality 2015 study by Mesch et al. [47] drew on a nationally representative sample of 968 US adults from October 2009 to understand how trust in both the federal government and local health care system to handle an outbreak of H1 N1 affected vaccine uptake. For both exposure measures, they found that trust was significantly associated with increased odds of vaccine uptake (trust in federal government: OR: 1.58, 95% CI: 1.10–2.26, *p* < 0.01; and trust in local health care system: 1.60, 95% CI: 1.04–2.45, *p* < 0.05).

A medium-quality 2021 study by Krupenkin [44] utilised a nationally representative sample of 1,004 US adults from October 2009 to examine the association between confidence in the US government and attitudes towards the safety of the H1 N1 vaccine. In doing so, it was found that feeling not so/not at all confident in the US government was significantly and negatively associated with attitudes towards the safety of the H1 N1 vaccine (regression coefficient: −2.226, SE: 0.855, *p* < 0.01), suggesting that, as a lack of confidence increased, perceptions of vaccine safety decreased. There were no significant associations found related to perceptions of vaccine safety among those who indicated they were somewhat/not so confident or somewhat/very confident in the government.

Finally, a high-quality, two-wave longitudinal survey of 601 French-speaking Swiss adults conducted during the 2009 H1 N1 outbreak by Gilles et al. [38] measured the association between trust in the WHO and pharmaceutical companies at baseline and both perceptions and uptake of the H1 N1 vaccine six months later. It was found that trust was significantly and positively associated with both having positive perceptions about the efficacy of the H1 N1 vaccine (β = 0.3, SE: 0.08, *p* < 0.001) and with vaccine uptake (β = 0.76, SE: SE: 0.21, *p* < 0.001) at follow-up.

#### Childhood vaccines

Four studies examined associations between populist-aligned attitudes and views on/uptake of childhood vaccines. In a medium-quality 2016 study of 1,253 parents of school children in Colorado, Massachusetts, Missouri and Washington by Lee et al. [45], the effect of trust in government and healthcare providers was measured in relation to both beliefs about and uptake of childhood vaccination. Adjusting for income, education, race, religiousness, and age, it was found that distrust in government was associated with increased the odds of believing that immunisations do more harm than good (OR: 1.95, 95% CI: 1.38–2.74, *p* < 0.01), as did a distrust in healthcare providers (OR: 2.03, 95% CI: 1.45–2.81, *p* < 0.01). The study also found that distrust in government and healthcare providers was significantly associated with higher odds of a parent not fully vaccinating their child (OR: 1.97, 95% CI: 1.45–2.67, *p* < 0.01; and OR: 2.18, 95% CI: 1.63–2.92, *p* < 0.01; respectively).

A medium-quality 2019 study of 575 Italian parents of children aged 1–5 years by Bianco et al. [30] examined the association between several variables related to populist-aligned beliefs and both child vaccination hesitancy and child vaccination refusal. It was found that agreement with the belief that infant vaccinations are primarily a money-making operation for pharmaceutical companies was significantly associated with increased odds of child vaccination refusal (AOR: 1.59, 95% CI: 1.01–2.51, *p* = 0.045), while trust in one’s paediatrician regarding information about vaccines was significantly associated with decreased odds of child vaccination refusal (AOR: 0.56, 95% CI: 0.32–0.96, *p* = 0.036).

A low-quality study of 972 Italian mothers of pre-school children aged 0–6 years published in 2020 by Selleri and Carugati [54] used multiple correspondence analysis to test associations between belief in conspiracy theories indicative of a distrust in pharmaceutical companies and agreement with the statement: “Vaccinating children is a private choice of parents: healthcare authorities do not have to intervene.” It was found that belief in these conspiracy theories was significantly associated with agreement that vaccinating children is a parent’s private choice (correlation coefficient: 0.399, *p* < 0.001). The study also examined the association between trust in science and attitudes towards childhood vaccination, finding a significant and positive relationship (0.213, *p* < 0.001), suggesting that as trust in science increased, so did positive attitudes towards childhood vaccination.

Finally, a medium-quality 2022 study by Aechtner and Farr [28] used data from 1,287 adult participants in the Australian Survey of Social Attitudes (AuSSA), finding a significant and positive association between confidence in Australia’s Federal Parliament and belief in the effectiveness of childhood vaccines (β: 0.161, 95% CI: 0.001–0.002, *p* < 0.000).

#### Human papillomavirus (HPV)

Three studies explored the relationship between populist-aligned attitudes and attitudes towards and/or uptake of the human papillomavirus (HPV) vaccine. A medium-quality study from 2015 by Hamada et al. [40] sampled 1,407 mothers of daughters aged 13–16 years across Fukuoka prefecture in Japan to examine associations between mothers’ trust in the government’s handling of vaccinations and their daughter’s HPV vaccination status. Compared to mothers with no trust in the government, those who trusted the government were significantly more likely to have had their daughters vaccinated against HPV (OR: 4.49, 95% CI: 3.17–6.37, *p* < 0.001), an outcome that remained after adjustment for educational background, annual household income, marital status and employment status (AOR: 2.40, 95% CI: 1.44–3.86, *p* < 0.001).

A medium-quality 2023 study by Frietz et al. [37] tested associations between trust in government related to vaccines and HPV vaccine intention as well as uptake among a sample of 602 predominantly Hispanic adults living in the US-Mexico border town of El Paso, Texas. It was found that trust in government was significantly and positively associated with HPV vaccine intention (β: 0.31, 95% CI: 0.22–0.43, *p* < 0.001), but was not significantly associated with HPV vaccine uptake.

Finally, in a medium-quality 2023 study of 870 German and Austrian adults, Kohler et al. [42] tested the association between holding science-related populist beliefs (based on conceptions of who constitutes ordinary people, conceptions of who constitutes the academic elite, demands for decision making sovereignty and demands for truth-speaking sovereignty) and uptake of the HPV vaccine. Adjusting for age, gender, education, health information seeking and health consciousness, the authors found no significant association between the two variables.

#### Measles, mumps and rubella (MMR)

Three studies tested associations between populist-aligned attitudes and attitudes towards and/or uptake of the measles, mumps and rubella (MMR) vaccine [46]. A low-quality 2015 study by Leonard drew on a sample of 243 US parents with at least one 6-year-old child to explore the association between trust in medical authorities (i.e., doctors and the government) and attitudes towards the MMR vaccine. In separate analyses that adjusted for parent gender, age and education, no significant association between trust in medical authorities and attitudes towards the MMR vaccine was found. There was also no evidence of moderation by age, gender or education.

A previously mentioned nationally representative study of 4,570 US adults conducted in 2021 by Krupenkin [44] tested the association between trust in government and attitudes towards the safety of the MMR vaccine. It was found that, while there was no significant association between those who felt somewhat/very confident in the US government and attitudes towards the safety of the MMR vaccine, those who felt somewhat/not very confident or not very/not at all confident in the US government were significantly more likely to have worse attitudes about MMR vaccine safety (Regression coefficient: −1.412, SE: 0.413, *p* < 0.01; and −2.795, SE: 0.419, *p* < 0.01; respectively).

Finally, a previously mentioned 2023 study of 870 German and Austrian adults by Kohler et al. [42] examined the association between holding science-related populist attitudes (based on conceptions of who constitutes ordinary people, conceptions of who constitutes the academic elite, demands for decision making sovereignty and demands for truth-speaking sovereignty) and MMR vaccination uptake. It was found that holding such attitudes was associated with significantly lower odds of receiving the MMR vaccine (OR: 0.602, 95% CI:0.49–0.72, *p* < 0.001).

#### Seasonal influenza

Three studies explored the relationship between populist-aligned attitudes and attitudes towards and/or uptake of a seasonal influenza vaccine. A medium-quality 2009 study by Musa et al. [48] drew on a sample of 1,681 Black and White US seniors from Allegheny County, Pennsylvania to understand the association between trust in local health departments, the Centers for Disease Control and Prevention (CDC) and one’s own doctor and uptake of a flu shot in the last year. Adjusting for gender, age, education, marital status, self-reported health status and number of existing health conditions, the study found no associations.

A previously mentioned 2012 study by Frew et al. [36] tested the association between a scale measuring conspiracy beliefs about H1 N1/mistrust of H1 N1 information coming from the government and intention to receive a seasonal flu vaccine among 503 US adults. It found a significant association between lower scores on the conspiracy belief/government mistrust scale and intentions to receive the flu vaccine (OR: 1.64, 95% CI: 1.23–2.19, *p* < 0.05).

Finally, another previously mentioned 2023 study by Kohler et al. [42] examined the association between holding science-related populist beliefs (based on conceptions of who constitutes ordinary people, conceptions of who constitutes the academic elite, demands for decision making sovereignty and demands for truth-speaking sovereignty) and uptake of the flu vaccine among 870 German and Austrian adults. Adjusting for age, gender, education, health information seeking and health consciousness, the study found no association between science-related populist beliefs and uptake of the flu vaccine.

#### Miscellaneous vaccines

Three studies examined the association between populist-aligned attitudes and other types of vaccination. A medium-quality 2018 study by Baumgaertner et al. [29] assessed the effect of trust in health care providers and government medical experts on combined attitudes towards pertussis, measles, and influenza vaccination. Based on their nationally representative sample of 1,006 US adults, the authors found significant and positive associations between trust in both health care providers and government medical experts and more positive attitudes towards pertussis, measles, and influenza vaccination, with trust in health care providers showing a slightly stronger association (β: 0.27, p ≤ 0.05; and β: 0.19, p ≤ 0.05; respectively).

A low-quality 2023 study of 26,313 Japanese adults by Hori et al. [41] looked at the association between trust in government and intention to receive the mpox vaccine according to participant sex. Adjusting for age group, educational background, sexual orientation, working status, household income, COVID-19 vaccination status, and frequency of going to a brothel, it was found that trust in government was significantly associated with increased mpox vaccine intention among both males and females (prevalence ratio: 1.37, 95% CI: 1.29–1.45; and 1.35, 95% CI: 1.23–1.47; respectively).

Finally, a previously mentioned 2023 study of 870 German and Austrian adults by Kohler et al. [42] tested the association between holding science-related populist attitudes (based on conceptions of who constitute ordinary people, conceptions of who constitutes the academic elite, demands for decision making sovereignty and demands for truth-speaking sovereignty) and uptake of both the tick-borne encephalitis (TBE) and meningococcal disease (MD) vaccines. It found no association between science-related populist beliefs and TBE vaccination, however science-related populist beliefs were significantly associated with reduced odds of receiving the MD vaccine (OR: 0.833, 95% CI: 0.71–0.97).

#### General vaccination attitudes

Four studies explored associations between populist-aligned attitudes and attitudes towards vaccination generally. A medium-quality study by Kossowska et al. [43] from 2021 examined attitudes towards vaccination among two samples of Polish adults. In the first sample (*n* = 391), a mediation analysis was conducted to understand the associations between right-wing political ideology, trust in scientists and positive attitudes towards vaccination. It was found that trust in scientists was significantly associated with more positive attitudes towards vaccination (β: 0.24, 95% CI: 0.14–0.34, *p* < 0.001). However, this association disappeared when trust in scientists was included in the model as a mediator between right-wing political ideology and positive attitudes towards vaccination. In the second sample (*n* = 376), the authors again used a mediation analysis to test the association between the perception of scientists as members of the country’s elite and positive attitudes towards vaccines. In this analysis, a significant and negative association was found (β: −0.21, 95% CI: −0.33 – −0.10, *p* < 0.01), though this disappeared when negative perceptions of scientists were included in the model as a mediator between right-wing political ideology and positive attitudes towards vaccines.

A previously mentioned 2022 paper by Aechtner and Farr [28] drew on data from 1,003 Australian adults participating in the Wellcome Global Monitor (WGM), where significant and positive associations were found between both general trust in scientists, as well as trust in scientists to be open and honest about who is paying for their work, and belief in vaccine safety (β: 0.110, 95% CI: 0.035–0.269, *p* = 0.01; and β: 0.083, 95% CI: 0.003–0.196, *p* = 0.043, respectively).

A medium-quality paper published in 2023 by Wright et al. [57] examined the association between trust in government to act in the interest of citizens and anti-vaccination attitudes among a convenience sample of 1,050 Australian parents. Adjusting for age, sex and education level, it was found that there was a significant and negative association between trust in government and anti-vaccination attitudes (β: −0.22, SE: 0.02, *p* < 0.001), indicating that as trust in government increased, anti-vaccination attitudes decreased.

Finally, in a medium-quality paper by Stasiuk et al. [55], it was found that changes in trust in both physicians and science among 400 Polish internet-using adults between 2018 and 2020 were significantly associated with similar changes in attitudes towards vaccination generally, meaning as trust in either physicians or science decreased, so did positive attitudes towards vaccination (change in trust in physicians: β = 0.244, SE: SE: 0.059; *p* < 0.001; change in trust in science: β = 0.288, SE: 0.058; *p* < 0.001).

### Sexual and reproductive health care

Five included studies examined the association between populist-aligned attitudes and attitudes towards, or engagement with, sexual and reproductive health care, where it was generally found that such attitudes were associated with decreased support for this type of intervention. Of these five studies, four studies looked at outcomes related to abortion. A low-quality 2019 study by Prusaczyk et al. [52] drew on a convenience sample of 296 US adults to estimate the role of hostile sexism (measured via the hostile sexism sub-scale of the Ambivalent Sexism Inventory) on support for abortion [58]. It was found that hostile sexism was significantly associated with reduced support for abortion (β= ‒0.11, 95% CI: −0.23–0.01, *p* = 0.08).

A medium-quality 2022 study of Polish adults by Szczepańska et al. [56] used two nationally representative samples to examine the association between hostile and benevolent sexism (measured using the Ambivalent Sexism Inventory), as well as hostile and benevolent sexism and prejudice towards people with Down’s syndrome, and support for a 2020 ruling restricting access to abortion in cases of fetal malformations [58]. In the first model (*n* = 994), the authors found a significant association between hostile, but not benevolent, sexism and support for the new abortion restrictions (β: 0.40, SE: 0.05; 95% CI: 0.29–0.50; *p* < 0.001). In the second model (*n* = 432), the authors once again found a significant association between hostile, but not benevolent, sexism and support for the new abortion restrictions (β: 0.41, SE: 0.12; 95% CI: 0.17–0.64; *p* < 0.001), as well as a significant association between prejudice towards people with Down’s syndrome (β: 0.32, SE: 0.15; 95% CI: 0.02–0.61; *p* < 0.05) and support for the new abortion restrictions.

A high-quality repeat cross-sectional study by Cizmar et al. [32] used nationally representative data from the US in 2012 (*n* = 11,424), 2016 (*n* = 4,270) and 2020 (*n* = 15,729) to examine the association between hostile sexism (measured via the hostile sexism sub-scale of the Ambivalent Sexism Inventory) and several different positions on abortion, categorized as support if there is a clear need; support in the case of rape, incest or protecting the woman’s health; and being purely pro-life; all compared to being purely pro-choice [58]. In 2012, it was found that compared to a purely pro-choice stance, hostile sexism was significantly associated with support for abortion only in the case of rape, incest or protecting the woman’s health (coefficient estimate: 1.07, SE: 0.31, *p* < 0.001), as well as having a purely pro-life stance (1.09, SE: 0.41, *p* < 0.01). When the analysis was repeated for 2016, hostile sexism was found to be significantly associated with support for abortion only in the case of clear need (2.00, SE: 0.46, *p* < 0.001), in the case of rape, incest or protecting the woman’s health (2.77, SE: 0.41, *p* < 0.001), as well as among those with a purely pro-life stance (2.80, SE: 0.53, *p* < 0.001). Finally, in 2020, hostile sexism was found to be significantly associated with support for abortion only in the case of rape, incest or protecting the woman’s health (1.15, SE: 0.40, *p* < 0.01) and with a purely pro-life stance (1.97, SE: 0.63, *p* < 0.01). In a second set of analyses, the authors also examined the role of hostile sexism in predicting purely pro-life versus purely pro-choice attitudes related to abortion. While there was no significant association found for the 2012 data, in 2016 and 2020, hostile sexism was found to be significantly associated with a higher probability of being pro-life (2016: 2.26, SE: 0.59, *p* < 0.001; and 2020: 1.94, SE: 0.73, *p* < 0.01).

A medium-quality 2022 paper by Gothreau et al. [39] drew on two nationally representative samples of US adults to examine associations between hostile and benevolent sexism (measured using the Ambivalent Sexism Inventory) and attitudes towards abortion, as well as women’s access to birth control and the use of federal funding to support the Planned Parenthood Federation of America (the latter two outcomes in the case of sample 1 only) [49, 58]. Among the first sample (*n* = 1,400), the authors found a significant association between hostile sexism and more negative attitudes towards abortion, women’s access to birth control and attitudes towards the use of federal funding to support Planned Parenthood (− 0.080, SE: 0.024, *p* < 0.01; − 0.205, SE: 0.020, *p* < 0.01; and − 0.325, SE: 0.033, *p* < 0.01, respectively). Within this first sample, the authors also found a significant association between benevolent sexism and more negative attitudes towards abortion and the use of federal funding to support Planned Parenthood (− 0.068, SE: 0.026, *p* < 0.05; and; − 0.069, SE: 0.035, *p* < 0.10, respectively). Turning to the second sample (*n* = 4,207), the authors found a significant association between hostile sexism and more negative attitudes towards abortion (− 0.129, SE: 0.030, *p* < 0.01).

Finally, a medium-quality study published in 2008 by Clark et al. [33] examined the association between HIV conspiracy beliefs (which measured a lack of trust in government and the pharmaceutical industry) and uptake of highly active antiretroviral therapy (HAART) among 113 HIV-positive adults in Houston, Texas. Using univariate statistical analysis, associations were tested related to the effect of holding HIV conspiracy beliefs among: 1) those who had never taken HAART and those who had; 2) those not currently on HAART and those currently on HAART; 3) those with < 100% self-reported adherence to HAART and those with 100% self-reported adherence; 4) those with < 80% adherence to HAART based on pharmacy refill data and those with ≥ 80% adherence; and 5) those with a gap in care > 120 days and those without a gap in care > 120 days. The authors found no statistically significant associations. Adjusting for race/ethnicity and time since HIV diagnosis in a multivariable regression analysis, the authors also found no statistically significant associations between holding HIV conspiracy beliefs and any of the patterns of HAART uptake.

### Preventive health care

Three included studies examined the association between populist-aligned attitudes and the use of preventive health care, where it was generally found that such attitudes were associated with decreased uptake of this type of intervention. A medium-quality study published in 2018 by Ojikutu et al. [49] drew on a sample of 855 Black US adults aged 18–50 years who were HIV-negative in order to assess the association between measures of trust on willingness to use pre-exposure prophylaxis (PrEP) to prevent against HIV transmission via sex or injection drug use [59]. As part of their analysis, the authors first tested the association between having trust in medical doctors (as compared to having a little trust or no trust) and willingness to use PrEP, finding no association among either all participants or those categorised as high risk. Second, they tested the association between HIV conspiracy beliefs (used to represent a lack of trust in government around HIV-related issues) and willingness to use PrEP, finding that holding HIV conspiracy beliefs was significantly associated with increased the odds of PrEP use among all participants (OR: 1.3, 95% CI: 1.1–1.5, *p* = 0.0016), but not among those categorised as high risk.

A medium-quality study published in 2019 by Powell et al. [50] drew on a sample of 610 Black US adults aged 20 years or older attending barber shops and academic institutions/events in Michigan, Georgia, California, and North Carolina in order to test the association between medical mistrust and delays in preventive health screening. Unadjusted analysis found that medical mistrust was significantly associated with increased delays in routine check-ups, blood pressure screening and cholesterol testing (OR: 2.76, 95% CI: 1.70, 4.47, *p* < 0.0001; OR: 2.50, 95% CI: 1.49–4.19, *p* < 0.0001; and OR: 1.45, 95% CI: 0.81–2.60, *p* = 0.22, respectively). Following adjustment for age, recruitment site, region, education, income, marital status, health insurance status, usual source of care, self-rated health status, chronic conditions and depressive symptoms, however, only the associations between medical mistrust and routine checkups and blood pressure screening remained statistically significant (AOR: 2.87, 95% CI: 1.45–5.71, *p* < 0.001; and 2.82, 95% CI: 1.31- 6.05, *p* < 0.01; respectively).

Finally, a previously mentioned 2009 study by Musa et al. [48] of 1,681 Black and White adults aged 65 + from Allegheny County, Pennsylvania examined the association between trust in local health departments, the CDC and one’s own doctor, and the uptake of a prostate-specific antigen (PSA) test for men, a mammogram for women, and a routine check-up for all participants. It was found that only trust in one’s own doctor was significantly associated with increased uptake of all outcomes (i.e., a PSA test in the last year among male participants: OR: 8.59, 95% CI: 2.66–27.68, p ≤ 0.001; a mammogram in the last two years for female participants: OR: 3.97, 95% CI: 1.17–13.55, p ≤ 0.05; and a routine check-up in the last year for all participants: OR: 3.04, 95% CI: 1.02–9.05, p ≤ 0.05).

### Disease screening

Two included studies examined the association between populist-aligned attitudes and disease screening, with a particular focus on HIV testing. Despite the small number of studies focused on this type of intervention, preliminary evidence supports the association between populist-aligned attitudes and HIV testing. A low-quality study published in 2013 by Ford et al. [35] tested the association between mistrust in government and HIV testing in the last 12 months among a socially vulnerable, racially/ethnically diverse group of 226 adults aged 50 or older living in Los Angeles. Adjusting for demographic factors, HIV risk, AIDS conspiracy beliefs and usual place of health care, they found that government mistrust was significantly associated with reduced HIV testing (AOR: 0.43, 95% CI: 0.26–0.73).

Another low-quality study published in 2017 by Fleming et al. [34] recruited 400 male clients of female sex workers (FSWs) at the San Diego-Tijuana border between the US and Mexico to test the association between misogynistic attitudes and ever having been tested for HIV. Using backwards stepwise multiple logistic regression and adjusting for age, the authors found that higher scores on a misogyny scale was significantly associated reduced odds of ever having been tested for HIV (AOR: 0.31, 95% CI: 0.11–0.84, *p* = 0.02).

### Non-pharmaceutical infection control measures

Finally, two studies examined the association between populist-aligned attitudes and adherence to non-pharmaceutical infection control measures. Again, despite the limited number of studies in this area, preliminary evidence supports the association between populist-aligned attitudes and adherence to such measures. A previously mentioned nationally representative study of 1,010 Italian adults published in 2011 by Prati et al. [51] assessed the association between trust in government authorities, trust in the media, trust in the Ministry of Health and trust in medical science, and compliance with a variety of recommended preventive measures during the 2009 H1 N1 influenza pandemic. It was found that trust in government authorities was significantly and positively associated with increased adherence to recommendations to use tissues while sneezing (AOR: 1.1, 95% CI: 1.0–1.2; *p* < 0.05). Trust in the media was significantly associated with increased adherence to recommendations to clean objects (AOR: 1.2, 95% CI: 1.1–1.3, *p* < 0.05), wash hands (AOR: 1.2, 95% CI: 1.1–1.2, *p* < 0.05), use tissues while sneezing (AOR: 1.1, 95% CI: 1.1–1.2, *p* < 0.05), and socially distance (AOR: 1.1, 95% CI: 1.1–1.2, *p* < 0.05). Trust in the Ministry of Health was similarly significantly associated with increased adherence to recommendations to clean objects (AOR: 1.1, 95% CI: 1.1–1.2, *p* < 0.05), wash hands (AOR: 1.1, 95% CI: 1.1–1.2, *p* < 0.05), use tissues while sneezing (AOR: 1.1, 95% CI: 1.0–1.1, *p* < 0.05), and socially distance (AOR: 1.1, 95% CI: 1.0–1.1, *p* < 0.05). Trust in medical science was only significantly associated with increased adherence to recommendations about cleaning objects (AOR: 1.1, 95% CI: 1.0–1.2, *p* < 0.05).

Finally, a previously mentioned longitudinal survey of 601 French-speaking Swiss adults conducted during the 2009 H1 N1 outbreak by Gilles et al. [38] examined the association between trust in the WHO and pharmaceutical companies at baseline and attitudes towards preventive measures against H1 N1 six months later. It was found that trust in these institutions was significantly associated with more positive perceptions about the efficacy of preventive measures such as handwashing (β = 0.17, SE: 0.06, *p* < 0.01) and wearing a mask (β = 0.22, SE: 0.08, *p* < 0.01).

## Discussion

### Summary of key findings

Overall, the findings from this evidence synthesis suggest that, among people living in high-income countries, different aspects of populist-aligned attitudes are generally associated with negative attitudes towards or reduced engagement with public health interventions addressing health areas other than COVID-19. The findings of this paper both support and substantially extend the findings from our previous syntheses, which suggested a similar finding for interventions aimed mainly at addressing COVID-19 [17, 18]. Together, the present findings and our previous syntheses on this topic also suggest there is merit in our argument that a breadth of populist-aligned views are of importance in the receipt of public health interventions.

Regarding vaccination, across the 21 included studies, it was generally found that a lack of trust in elite institutions or actors such as government, scientists, pharmaceutical companies and the health care system was significantly associated with more negative views on, and reduced uptake of, vaccines for both adults and their children. This link between populist-aligned attitudes and vaccine hesitancy is in line with previous research in this area. For example, in a 2019 study of national-level data across western Europe, Kennedy [1] found a significant association between the percentage of people in a country who voted for populist parties and those who believed that vaccines were neither important nor effective. A 2022 study of vaccine hesitancy across the European Union by Stoeckel et al. [60] (excluded from our review due to pooled analysis with non-OECD countries) similarly found that vaccine hesitancy was associated with populist attitudes, and specifically anti-elite worldviews.

Regarding sexual and reproductive health care, of the four studies that examined the association between populist-aligned hostility towards outgroups and support for restricted abortion access, the evidence consistently suggested a significant and positive relationship [32, 39, 52, 56]. This was especially true when it came to measuring the association of hostile sexism and support for abortion restrictions, though one study conducted in Poland also found a significant association between prejudice towards people with Down’s syndrome and support for abortion restrictions. In the case of the US, data from nationally representative, repeat cross-sectional population samples taken in 2012, 2016 and 2020 provided evidence of the increasing association over time between hostile sexism and identifying as pro-life rather than pro-choice [32]. This latter finding is particularly interesting given the ways in which the survey years overlap with the rise of Donald Trump, whose first tenure as US President from 2017 to 2021 was seen as pivotal in paving the way for the Supreme Court’s 2022 decision to overturn the Roe versus Wade judgment which had previously protected women’s right to an abortion under the US Constitution [61]. This overlap with President Trump’s time in office may also be relevant for the included US study that looked at the association in 2016 and 2018 between hostile sexism and reduced support for women’s access to birth control and the provision of federal funding for Planned Parenthood.

Among the three studies that examined the role of populist-aligned beliefs in the uptake of preventive care, though the types of preventive care largely differed across the studies, there was consistent evidence of the importance of trust in medical professionals in the uptake of activities such as routine check-ups, blood pressure screening, annual PSA testing,^2^ and bi-annual mammograms [48, 50]. These studies also suggested the significant and harmful impact of medical mistrust on uptake of preventive health care interventions for racial/ethnic minorities. This latter finding is in line with our previous syntheses of public health interventions addressing COVID-19 and uptake of the HPV vaccine for young girls, as well as with recent studies that have examined the role of medical mistrust in reducing uptake of smear tests among Indigenous populations in North America [17, 18, 63–66]. Among included studies focused on preventive care, it was also found that increased government mistrust around HIV-related issues was associated with increased use of PrEP to prevent against HIV transmission [49]. While this may at first seem like a counter-intuitive finding, several of the statements used to assess mistrust in government in this study (i.e., ‘There is a cure for HIV but the government is withholding it from the poor’ and ‘The medicine that doctors prescribe to treat HIV is poison’) potentially indicate a natural desire among participants to protect themselves against the perceived actions of an elite they do not trust. Overall, however, given the limited number of included studies examining the association between populist-aligned attitudes and uptake of preventive care, and the small number of interventions examined, these results should be treated with caution.

Only two of the included studies focused on disease screening, both examining uptake of HIV testing among vulnerable sub-samples of the US population. Although these studies examined different aspects of populist-aligned attitudes (i.e., trust in government and hostility towards women), both found that holding such views was significantly and negatively associated with HIV testing [34, 35].

Lastly, while only two studies examined the relationship between populist-aligned attitudes (measured in terms of trust in elite institutions and actors such as the government, the media, the Ministry of Health, medical science, the WHO and the pharmaceutical industry) and adherence to various non-pharmaceutical infection control measures during the 2009 H1 N1 pandemic, these studies both reported significant associations [38, 51]. These findings echo similar ones from across a large number of studies related to the association between distrust in various elite institutions and actors and reduced adherence to preventive guidance during the COVID-19 pandemic [67–72].

Based on the GRADE framework, the confidence in the findings of this review can be considered, respectively, moderate, moderate, very low, very low and very low for attitudes towards/uptake of: vaccination; sexual and reproductive health care; preventive health care; disease screening; and non-pharmaceutical infection control measures.

### Limitations

The findings from this study should be viewed in light of four key limitations. First, as with the wider systematic review from which this synthesis originates, while our aim was to develop clear inclusion criteria and search terms that align with the core aspects of populism as it is commonly understood in the existing literature, this did not require authors to explicitly use terms such as’populism’ or’populist’ when describing their measures. As described in our methods, we made this decision because’populism’ is a contested socio-political construct often used to make (largely critical) assessments about those holding such beliefs and as such, we aimed to avoid biasing our inclusion towards studies taking a particular position on this [11]. While this approach has allowed us to find evidence of the role that trust in elite institutions and actors and hostility towards various out-groups play in attitudes towards/uptake of a number of different public health interventions, it may be that not all of the exposure measures in the included studies are best understood as indicating populist ideas. This may be the case, for example, regarding measures of medical mistrust among racial/ethnic minorities and other marginalised communities. Nonetheless, the fact that our findings suggest that measures of views aligned with different facets of populism, such as distrust in elites and hostility towards out-groups, all tend to be associated with more negative attitudes towards or reduced engagement with public health interventions does suggest that it is useful to bring together the evidence from different studies in this way within the wider framework of populism.

A second limitation of this study is that not all of the included studies we synthesised adjusted for important potential confounders (see Table 1 for the list of adjustments made in each study). Despite this, however, even in the small number of studies where this was the case, the overall findings related to how people holding populist-aligned views engage less with public health interventions remain salient.

Thirdly, nearly all of the included studies were cross-sectional. This reduces our ability to determine if associations found are suggestive of populist-aligned views causing reduced acceptability of or engagement with public health interventions. Despite this, however, we argue that the presented evidence that those holding populist-aligned views tend to engage less with such interventions is extremely important for policy regardless of causal directions. Finally, for some topics, such as adherence to various non-pharmaceutical infection control measures, there were few studies.

## Conclusions

### Implications for research and policy

Evidence on the influence of populist-aligned attitudes on engagement with public health interventions beyond those aimed at addressing COVID-19 is emerging. Further research is indicated in areas such as sexual and reproductive health care, disease screening and preventive care. Such research would benefit from valid measures from across the constellation of views associated with the term ‘populism’, as well as longitudinal study designs aimed at exploring the causal inter-relationship between such views and engagement with public health interventions. It would also be useful to explore how different views linked to populism co-occur using factor analysis or latent class analysis.

In terms of implications for policy, the evidence presented here adds support to our previous findings on the importance of prioritising trust-building between the populations of high-income countries and their respective political, scientific and medical establishments to encourage greater acceptance and uptake of important public health interventions. The present findings also uniquely point to the need for policymakers to devise mitigation strategies that can overcome the negative impact of populist-style hostility towards out-groups as it relates to support for more highly politicised public health interventions, including access to abortion and family planning services. One way to do this could be to draw on empathy-centred approaches to health promotion [73]. These present public health interventions as being used or one day potentially being used by people whom individuals holding such populist-aligned attitudes care about as a means to personalise rather than politicise engagement with these interventions based on empathy rather than psychological distancing. Another way would be to engage with communities earlier in the development or delivery of public health interventions through PPIE so that their concerns are addressed and interventions rendered more acceptable [74].

## Supplementary Information

Supplementary Material 1.

## Data Availability

All data are already in the public realm because this is a systematic review of published evidence.

## Abbreviations

AOR: Adjusted Odds Ratio
AuSSA: Australian Survey of Social Attitudes
BeSST: Behavioural and Social Sciences Team
CDC: Centers for Disease Control and Prevention
DEI: Diversity, Equity and Inclusion
DHSC: Department of Health and Social Care
FSW: Female Sex Workers
GRADE: Grading of Recommendations, Assessment, Development, and Evaluations
HAART: Highly Active Antiretroviral Therapy
HIV: Human Immunodeficiency Virus
HPV: Human Papillomavirus
MD: Meningococcal Disease
MESH: Medical Subject Headings
MMR: Measles, Mumps and Rubella
NIHR: National Institute for Health and Care Research
OECD: Organisation for Economic Co-operation and Development
OHID: Office for Health Improvement and Disparities
OLS: Ordinary Least Squares
OR: Odds Ratio
PPIE: Patient and Public Involvement and Engagement
PrEP: Pre-exposure Prophylaxis
PRISMA: Preferred Reporting Items for Systematic Reviews and Meta-Analyses
PSA: Prostate-specific Antigen
RR: Relative Risk
TBE: Tick-borne Encephalitis
WGM: Wellcome Global Monitor
WHO: World Health Organization

## Footnotes

1 For an overview of the study characteristics of all 238 studies included in the wider systematic review, see Appendix 3.

2 It should be noted that guidance around routine PSA testing as a form of preventive care is not the same in every jurisdiction (see, for example, the UK) [62]. As such, this finding and how it relates to the impact of populist-aligned attitudes on engagement with public health interventions should be taken as context-specific.

## Declarations

Ethics approval and consent to participate.

Not required.
