# Correction: How populist-aligned views affect receipt of non-COVID-19-related public health interventions: a systematic review of quantitative studies

**DOI:** 10.1186/s12889-026-29536-x

**Published:** 2026-09-23

**Authors:** Kaitlin Conway‑Moore, Jack M. Birch, Alison R. McKinlay, Fiona Graham, Emily Oliver, Clare Bambra, Michael P. Kelly, Chris Bonell

**Affiliations:** 1 Department of Public Health, NIHR Policy Research Unit Behavioural and Social Sciences, Environments and Society, London School of Hygiene and Tropical Medicine, London, WC1H 9SH UK; 2 Faculty of Medical Sciences, NIHR Policy Research Unit Behavioural and Social Sciences, Population Health Sciences Institute, Newcastle University, Newcastle Upon Tyne, UK; 3 Department of Clinical, Educational and Health Psychology, NIHR Policy Research Unit Behavioural and Social Sciences, Centre for Behaviour Change, University College London, London, UK; 4 Department of Public Health and Primary Care, Cambridge Public Health, University of Cambridge, Cambridge, UK

**Correction: BMC Public Health 25, 2075 (2025)**

**https://doi.org/10.1186/s12889-025-23265-3**

Following publication of the original article [1], an error was identified in the Measles, mumps and rubella (MMR) section.

The sentences previously read:

In separate analyses that adjusted for parent gender, age and education, no significant association between trust in medical authorities and attitudes towards the MMR vaccine was found. There was also no evidence of moderation by age, gender or education.

The corrected sentences reads:

The study found a significant positive association between trust in medical authorities and attitudes towards the MMR vaccine (R² = 0.10, F(1,235) = 26.39, *p* < 0.001). Subsequent moderation analyses found no evidence that this association was moderated by parent age, gender, or education.

This error has no implications for the overall conclusions of the review as reported in the abstract or discussion section.
